# Validation of epigenetic mechanisms regulating gene expression in canine B-cell lymphoma: An *in vitro* and *in vivo* approach

**DOI:** 10.1371/journal.pone.0208709

**Published:** 2018-12-11

**Authors:** Silvia Da Ros, Luca Aresu, Serena Ferraresso, Eleonora Zorzan, Eugenio Gaudio, Francesco Bertoni, Mauro Dacasto, Mery Giantin

**Affiliations:** 1 Department of Comparative Biomedicine and Food Science, University of Padova, Padova, Italy; 2 Department of Veterinary Sciences, University of Turin, Turin, Italy; 3 Università della Svizzera italiana, Institute of Oncology Research, Bellinzona, Switzerland; 4 Oncology Institute of Southern Switzerland, Bellinzona, Switzerland; University of South Alabama Mitchell Cancer Institute, UNITED STATES

## Abstract

Despite canine B-cell Lymphoma (BCL) representing the most common haematological tumour, epigenetic events driving development and progression are scarcely known. Recently, canine Diffuse Large BCL (DLBCL) DNA methylome by genome-wide CpG microarray has identified genes and pathways associated to pathogenesis. To validate data previously obtained by array analysis, the CLBL-1 cell line was used and the *HOXD10*, *FGFR2*, *ITIH5* and *RASAL3* genes were selected. CLBL-1 cells were treated with two hypomethylating drugs (HDs; IC_50,_ 50% inhibitory concentration), i.e. azacytidine and decitabine (DEC), either alone or in combination with three histone deacetylase inhibitors (HDACis; IC_20_), i.e. valproic acid, trichostatin and vorinostat. Following the incubation with both HDs, an overall decrease of promoter methylation was highlighted, thus confirming target genes hypermethylation. The highest mRNA restoration was observed following the exposure to HDs combined with HDACis, and mostly with valproic acid. Contrasting results were only obtained for *RASAL3*. An *in vivo* confirmation was finally attempted treating Nod-Scid mice engrafted with CLBL-1 cells with DEC. Although DEC did not arrest tumour growth, target genes promoter methylation was significantly reduced in DEC-treated mice *vs* controls. Overall, this work demonstrates that CLBL-1 cell line represents a reliable *in vitro* model to validate the methylation-dependent silencing of key genes for BCL; moreover, it may be useful for xenograft models in mice, despite its aggressive behaviour. In future, functional studies will be performed to deepen the role of selected genes on BCL pathogenesis and progression, and their methylation-dependent mechanism of regulation.

## Introduction

DNA methylation and acetylation of nucleosomal histones are probably the most investigated epigenetic modifications, potentially leading to gene expression alteration and chromatin structure re-building in cancer [1–2]. Hypermethylation of CpG islands in promoter regions of tumour suppressor genes (TSGs) is known to cause inhibition of transcription factors binding [1, 3–5], whereas aberrant histone deacetylation can reduce gene expression through the re-building of chromatin structure [2, 6]. In addition, these two epigenetic mechanisms are likely to collaborate. Biologically, methyl CpG binding proteins (MBPs) bind to methylated CpG sites in gene promoters; then co-repressors such as histone deacetylases, methyltransferases and chromatin remodelling factors are recruited. This mechanism results in a reduction of transcription factors recognition [3, 7–11].

The contribution of several epigenetic mechanisms in human B-cell lymphoma (BCL) has been recently demonstrated [12]. Canine lymphoma shows clinical presentation, biology, and treatment approaches overlapping with the human counterpart [13–19]. Considering these similarities, previous studies investigated TSGs methylation in dog [20–23], increasing the knowledge beyond canine BCL pathogenesis and progression, and opening the rationale to transfer hypomethylating drugs (HDs) and histone deacetylases inhibitors (HDACis) into veterinary oncology. While drugs targeting epigenetic regulators in human oncology are widely used [2, 3, 6, 8–10], few data on the use of HDs and HDACis are actually available in dog [24, 25]. Conversely, their efficacy in canine *in vitro* models has already been shown [21–23, 26, 27].

In the present study, we used a validated canine BCL *in vitro* model, i.e. the CLBL-1 cell line [28], to investigate the methylation-dependent regulation of four TSGs, namely the Homeobox D10 (*HOXD10)*, the Fibroblast Growth Factor Receptor 2 (*FGFR2)*, the Inter-Alpha-Trypsin Inhibitor Heavy Chain Family Member 5 (*ITIH5*) and the RAS Protein Activator Like 3 (*RASAL3*); these genes have been shown to be hypermethylated in canine diffuse large B-cell lymphoma (DLBCL) [29]. First, we assessed the effects of HDs and HDACis on methylation and transcription by an *in vitro* experiment; second, we evaluated the anti-tumour and demethylating activity of DEC in NOD-Scid mice engrafted with CLBL-1 cells.

## Materials and methods

### Cell line

CLBL-1 cells, isolated from the peripheral lymph node of a dog with confirmed stage IV DLBCL [28], were maintained in T25 or T75 flasks under humidified 5% CO_2_ atmosphere, at 37°C. Cells were grown in RPMI 1640 medium (Gibco, Thermo Fisher Scientific Waltham, Massachusetts, USA), supplemented with 10% foetal bovine serum (Gibco, Thermo Fisher Scientific Waltham, Massachusetts, USA), 2 mM L-glutamine and 1% penicillin/streptomycin solution (10,000 UI/mL, Thermo Fisher Scientific Waltham, Massachusetts, USA).

### Products and solutions

Azacytidine (AZA), decitabine (DEC), valproic acid (VA), trichostatin A (TSA) and vorinostat (SAHA) were purchased from Sigma-Aldrich (Milan, Italy). Stock solutions of DEC, TSA and SAHA were prepared in DMSO and stored at -20°C. AZA and VA were prepared in RPMI medium immediately before use.

### HDs and HDACis cytotoxicity assays

CLBL-1 cells were seeded in a 96-well flat bottom plate (Sarstedt Italia, Verona, Italy) at a concentration of 2×10^4^ cells/well (180 μL). HDs and HDACis (20 μL) were added directly into each well. The range of concentrations used for the cytotoxicity screening was 0.2–50 μM and 0.002 nM—40 μM for AZA and DEC, respectively. Concentrations between 0.10 nM—0.165 μM, 0.0001–40 μM and 0.02–6 mM were chosen for TSA, SAHA and VA, respectively. Additional wells were exposed either to the vehicle (dimethyl sulfoxide, DMSO, 0.1% final concentration) for DEC, TSA and SAHA or to the cell culture medium (AZA and VA). Cells were incubated for 72 h with HDs, while HDACis were added only in the last 24 h of the experiment. The incubation time of 72 h was established based on the cell line doubling time (31 hours) [28], and guaranteed at least two replicative cycles, necessary for allowing the explication of HD effects. Conversely, for HDACis a shorter incubation time (24 h) was considered sufficient for maximizing the effects on gene expression, based on preliminary investigations.

Due to its chemical instability, AZA dilutions were freshly daily prepared. At the end of the experiment, 20 μL of CellTiter-Blue Reagent (Alamar Blue, Promega, Madison, USA) was added to each well, and the fluorescence was measured at 560 nm as excitation wavelength and 590 nm as emission wavelength by using a VICTOR X4 Multilabel Plate Reader (Perkin Elmer, Waltham, USA). Five separate experiments were executed and each concentration was tested in sextuplicate. The mean value of the % of mortality obtained from each individual cytotoxicity experiment was determined. The dose-response curve was obtained using GraphPad Prism 5 for Windows (GraphPad Software, San Diego, USA) and the drug concentration causing 50% reduction of cell viability (IC_50_ value) was calculated.

### Cell treatment

Cells were seeded at a concentration of 3×10^5^ cells/well in a 6-well flat bottom plate (Sarstedt Italia, Verona, Italy), and incubated for 72 h. HDs (300 μL of a stock solution 10X) were added directly onto each well after the cell suspension (2700 μL). HDACis (30 μL of a 100X stock solution) were added either in wells containing cells exposed to HDs than in untreated cells in the last 24 h of treatment. Likewise to cytotoxicity, AZA solution 100X were prepared every 24h. According to preliminary investigations, final concentrations corresponding to IC_50_ and IC_20_ values were used for HDs and HDACis, respectively. The IC_20_ value was calculated as follows: IC_20_ = (F/100-F)^1/H^ * IC_50_, where F is 20 and H is Hillslope.

Four independent experiments were performed. At the end of the treatment, cells were washed with PBS. Then, gDNA and total RNA were extracted using the DNeasy Blood & Tissue Kit and the RNeasy Mini Kit (Qiagen, Hilden, Germany), respectively, as per manufacturer’s instructions. Concentrations were measured with NanoDrop 1000 Spectrophotometer (Thermo Scientific, Waltham, Massachusetts, USA).

### Bisulfite conversion and Methyl Specific PCR (MSP)

Five hundred ng of gDNA were bisulfite converted following the protocol of MethylCodeBisulfite Conversion Kit (Invitrogen, Carlsbad, California, United States). The thermocycler Proflex PCR System (Life Technologies, Carlsbad, California, United States) was used for DNA denaturation at 95°C and bisulfite conversion at 64°C.

Four TSGs (*HOXD10*, *FGFR2*, *ITIH5* and *RASAL3*), characterized by an abnormal hypermethylation status [29] as well as a significant lower mRNA expression in DLBCLs compared to controls (S1 Fig), were selected as target genes. *RPL8* was included as negative control gene.

Methylation (Meth) and No Methylation (No Meth) primers for *HOXD10*, *FGFR2*, *ITIH5* and *RASAL3* are reported in [29]; for *RPL8* the following oligonucleotides have been used: forward (F) Meth: 5’- GTATCGGGTTTGCGGTC -3’; reverse (R) Meth: 5’- TACCTACTACCGAACGCGAC -3’; F No Meth: 5’- GTGTATTGGGTTTGTGGTT -3’; R No Meth: 5’- CCTACCTACTACCAAACACAAC -3’. Primers were designed on the CpG islands identified in the promoter region using Methyl Primer Express software v1.0 (Applied Biosystems, Foster City, CA) as previously described [30, 31]. In details, the DNA regions considered for the primer design were those recognized by probes of the previously used canine Agilent CpG microarray platform (GEO accession: GPL23069) [29]. Oligonucleotides were synthesized by Eurofins MWG Synthesis GmbH (Ebersberg, Germany). The quantitative Real-Time PCR (qPCR) amplification was carried out using the Power SYBR Green PCR Master Mix (Applied Biosystems, Foster City, CA) and Stratagene Mx3000P (Agilent Technologies Santa Clara, California, United States). Standard qPCR conditions were used except for *ITIH5*, for which Ct values were acquired at 74°C to eliminate primer dimers contribution to the fluorescence signal acquisition. Different concentrations of F and R primers of both Meth and No Meth were tested: 50/50, 300/300, 300/600, 600/300 and 600/600 nM. The specific amplification was checked loading MSP products in a 2% agarose gel. For each gene, the level of methylation was estimated by calculating the ratio of unmethylated to methylated assays as ΔCt (= Ct No Meth–Ct Meth), as previously described [32]. In case of absence of No Meth assay amplification, a Ct value of 40 was arbitrarily assigned to permit the ΔCt calculation. The appropriate concentrations of Meth and No Meth primer pairs were initially set up (S1 Table). The specificity of each primer pair was validated performing qPCR both with bisulfite converted and non-bisulfite converted gDNA, analysing the melting curves and loading the amplification products on a 2% agarose gel.

### Reverse transcription (RT) and qPCR

The RT (1 μg of total RNA) was performed by using the High Capacity cDNA Reverse Transcription kit (Life Technologies, Carlsbad, California, United States), according to the manufacturer’s instructions. For each target transcript and the negative control *RPL8*, gene-specific primers encompassing one intron were designed by using the Universal Probe Library (UPL) Assay Design Centre web service (Roche Diagnostics, Mannheim, Germany). Two previously published internal control genes (ICGs), i.e. *GOLGA1* and *CCZ1* [33, 34], were selected for this study, for the absence of a statistically significant modulation in treated *vs* untreated cells. Oligonucleotides were synthesized by Eurofins MWG Synthesis GmbH (Ebersberg, Germany) and are reported in S2 Table. The qPCR reaction was performed in a final volume of 10 μL, using 12.5 ng of cDNA, the Power SYBR Green PCR Master Mix (Life Technologies, Carlsbad, California, United States) and a Stratagene Mx3000P thermal cycler (Agilent Technologies, Santa Clara, California, United States). Samples were analysed in duplicate. Standard qPCR conditions were used, except for the analysis of *ITIH5*, for which Ct values were acquired at 78°C to eliminate primer dimers contribution to the fluorescence signal acquisition. Different concentrations of F and R primers were tested: 50/50, 50/300, 300/50 and 300/300 nM. The presence of specific amplification products was confirmed by dissociation curve analysis. For each qPCR assay, negative controls (with either total RNA or water as template) were run. Standard curves were obtained using the best performing primer combination and serial dilutions of cDNA from CLBL-1 cells or control lymph node. Each dilution was amplified in duplicate. The ΔΔCt method [35] was used to analyze gene expression results. All assays showed an acceptable efficiency (range 90% ÷ 110%), and a slope comprised between -3.6 and -3.1 (S3 Table).

### Xenograft experiment

NOD-Scid (NOD.CB17-Prkdcscid/NCrHsd) mice (five-six weeks of age, approximately 20 g body weight) were purchased from Harlan Laboratory. Mice maintenance and animal experiments were performed under institutional guidelines established for the Animal Facility of the Institute of Research in Biomedicine (IRB; Bellinzona, Switzerland) and with study protocols approved by the local Cantonal Veterinary Authority. Mice were subcutaneously engrafted with 15 x10^6^ CLBL-1 cells and divided into two experimental groups (n = 4). Starting with an average tumour volume of 800 mm^3^, mice underwent treatment either with the single agent DEC (diluted in water) or vehicle only. DEC was administered four times at the dose of 2 mg/kg (Day 1) and 4 mg/kg (Days 2, 3, 4) through i.p. injections. Engrafted mice were monitored daily, and treatments were performed without anaesthesia. Mice were sacrificed by inhalation of CO_2_ and with tumours smaller than 2000 mm^3^, according to guidelines reported by the Cantonal Veterinary Authority. At the sacrifice, tumours transplanted were collected and measured in size. Small fragments of about 100 mg each were stored at -80°C for subsequent gDNA and total RNA isolation. These samples were subjected to qPCR and MSP analysis of the four target genes and the negative control *RPL8*.

### Statistical analysis

Statistical analysis was performed using GraphPad Prism version 5.00 for Windows (GraphPad Software, San Diego, USA). Data referring to the drug-dependent variation in target gene expression were expressed as 2^-ΔΔCt^, and statistically analysed using unpaired T test or one-way analysis of variance (ANOVA) followed by the Tukey’s Multiple Comparison Test. A *P* value < 0.05 was considered as statistically significant.

## Results

### HDs and HDACis cytotoxicity

**Fig 1:**
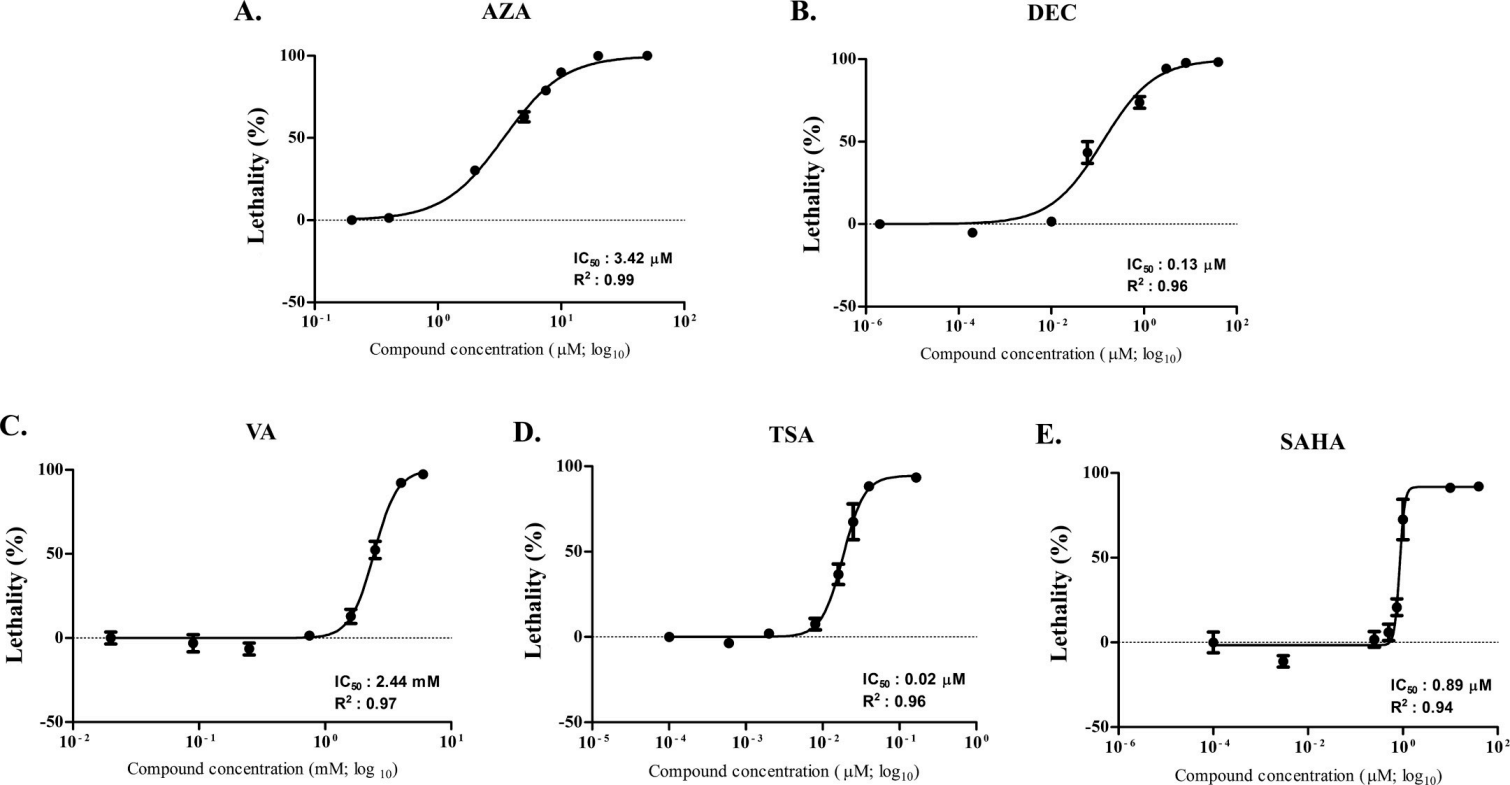
Dose-response curves (Alamar blue test). Relative IC_50_ and R^2^ values were obtained after incubation (72 or 24 h) of CLBL-1 cells with AZA (A), DEC (B), VA (C), TSA (D), SAHA (E).

All the HDs and HDACis were cytotoxic after 72h and 24h of incubation, respectively. Sigmoidal dose-response curves, relative IC_50_ values and corresponding linear regression coefficients (R^2^) for each drug are shown in Fig 1.

Concerning HDs (Fig 1A and 1B), the CLBL-1 cell line was more sensitive to DEC than AZA, with IC_50_ values equal to 0.13 and 3.42 μM, respectively. As to HDACis, TSA and SAHA (Fig 1D and 1E) were cytotoxic at lower concentrations (with IC_50_ values corresponding to 0.02 and 0.89 μM, respectively) if compared to VA (Fig 1C), for which the IC_50_ value was 2.44 mM. According to these cytotoxicity assays and further preliminary investigations (data not shown), the IC_50_ values for AZA and DEC, and the IC_20_ values for VA, TSA and SAHA, were selected for the subsequent experiments.

### MSP assays validation

The validation of MSP assays (agarose gel electrophoresis and melting curve analysis) for *HOXD10* and *FGFR2* is exemplified in S2–S4 Figs. PCR products obtained from the amplification of bisulfite-converted gDNA, non bisulfite-converted gDNA and blank with both Meth and No Meth assays were loaded on a 2% agarose gel (S2 Fig). *HOXD10* and *FGFR2* amplification was specific with both set of primers pairs, as only one amplicon of the expected size was detected (S2 Fig, lanes 1 and 4). Primer dimers, if present, were noticed only in non bisulfite-converted gDNA (lanes 2 and 5) and in the blank samples (lanes 3 and 6), where there was no possibility for their annealing with gDNA. However, the presence of dimers in negative controls is attributable to their relatively high or low GC% content. This result was confirmed by melting curve analysis, in which the negative controls (blank) and not bisulfite-treated gDNA were mainly characterized by primer dimers (grey and green lines), having a low melting temperature (72–74°C, S3 and S4 Figs). Conversely, the peaks on the right, characterized by a higher melting temperature (>78–80°C), were referable to the amplification of specific PCR products. Numerous peaks were caused by the presence of multiple amplicons of the same length, but characterized by a different C and T composition, for the coexistence of hemi-methylated DNA molecules.

### MSP analysis in CLBL-1 cells

**Fig 2:**
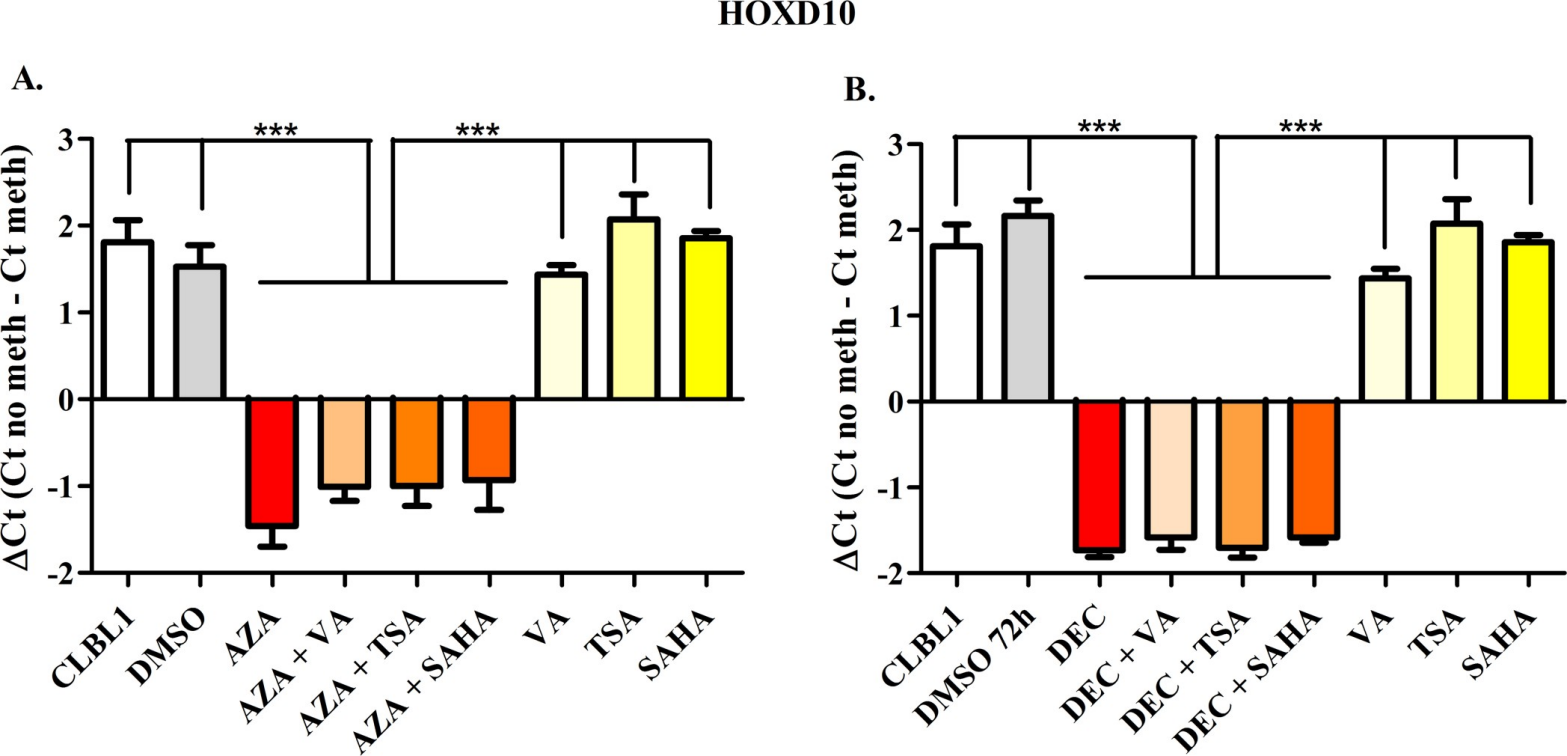
*HOXD10* Methyl Sensitive PCR after exposure to HDs (72 h) and HDACis (in the last 24 h of treatment), incubated alone or in combination. The effects of AZA or DEC, alone or in combination with HDACis are reported in panels A and B, respectively. Data are expressed as ΔCt (= Ct No Meth–Ct Meth), as means ± SEM. Statistical analysis: ANOVA + Tukey’s post-test. ***: *P* < 0.001.

**Fig 3:**
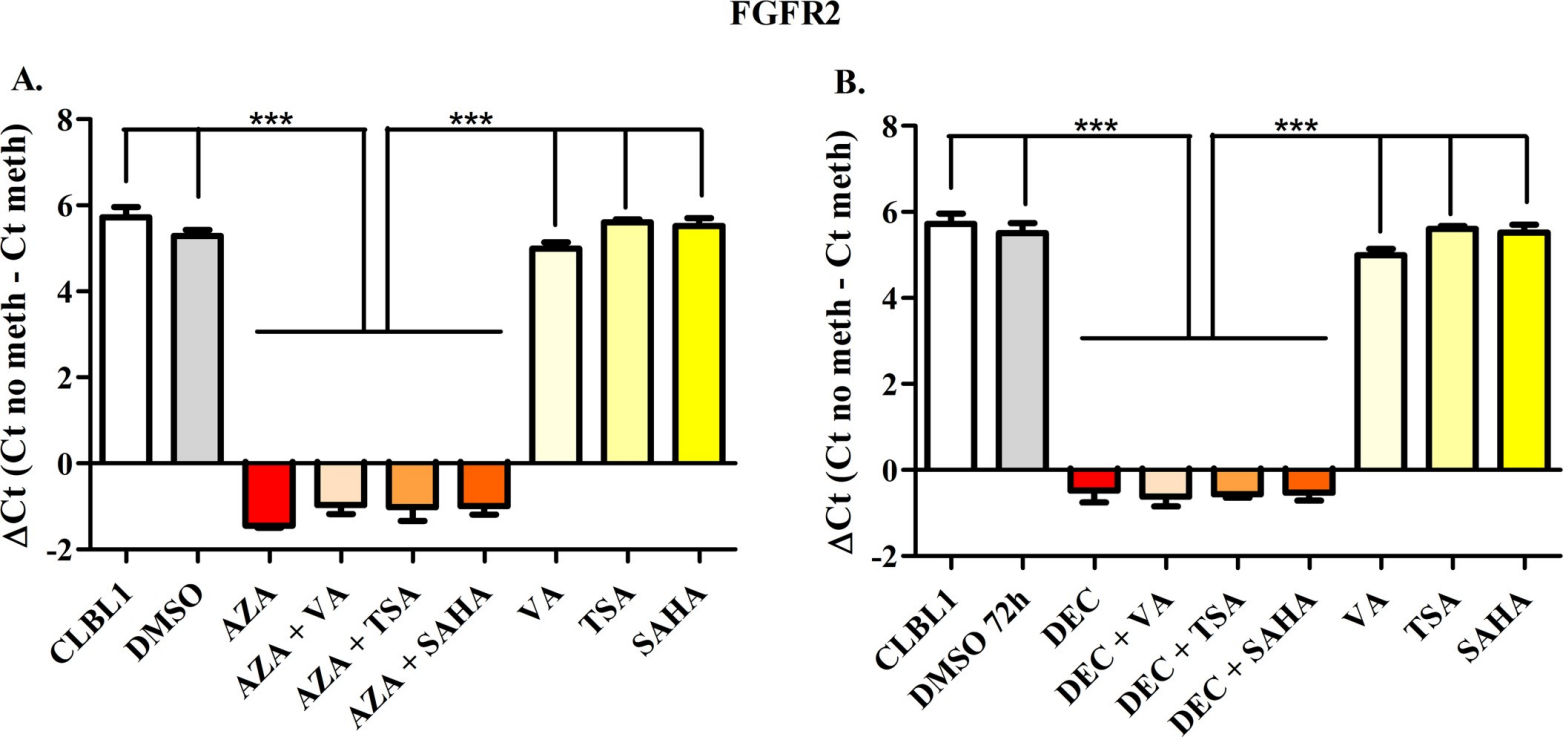
*FGFR2* Methyl Sensitive PCR after exposure to HDs (72h) and HDACis (in the last 24 h of treatment), incubated alone or in combination. The effects of AZA or DEC, alone or in combination with HDACis are reported in panels A and B, respectively. Data are expressed as ΔCt (= Ct No Meth–Ct Meth), as means ± SEM. Statistical analysis: ANOVA + Tukey’s post-test. ***: *P* < 0.001.

**Fig 4:**
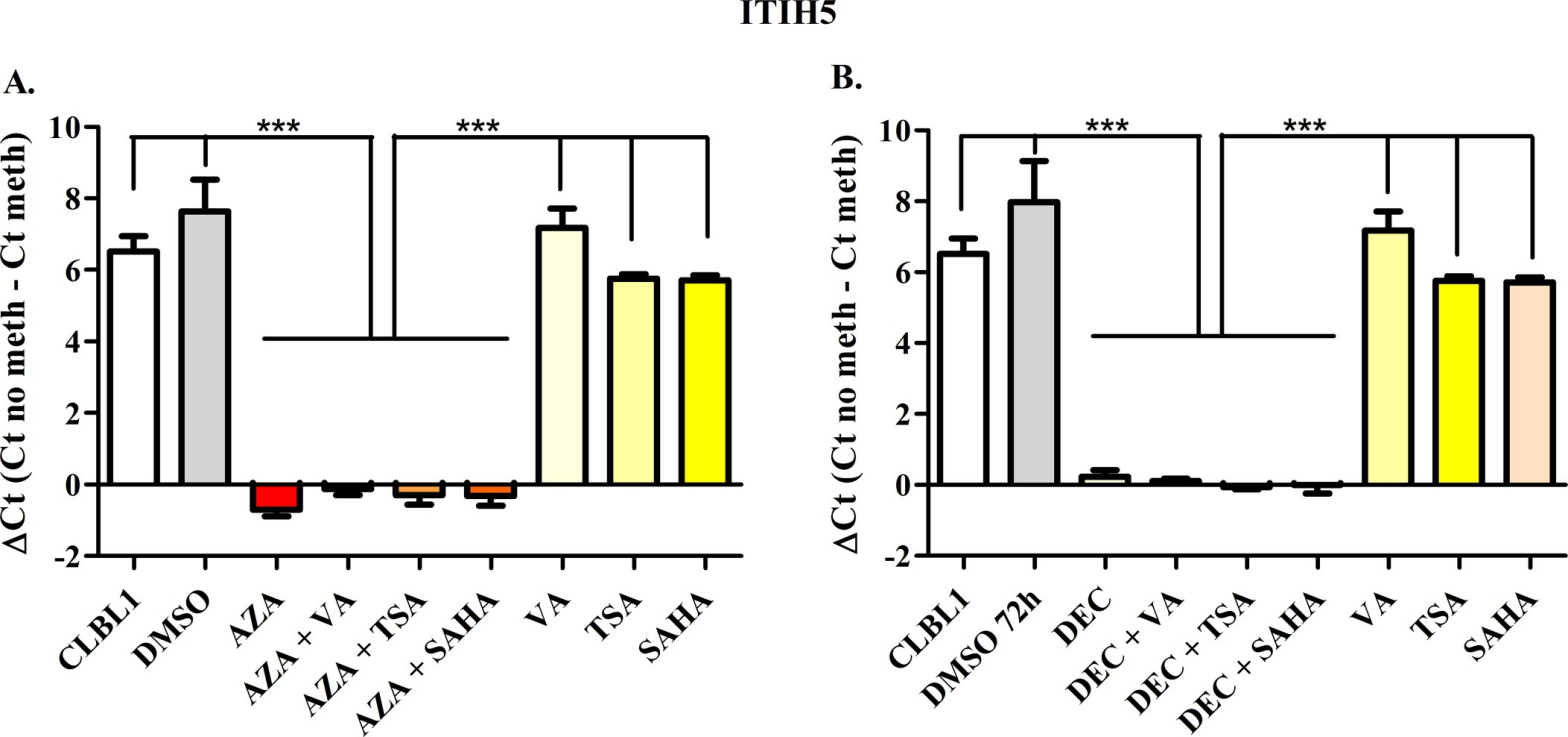
*ITIH5* Methyl Sensitive PCR after exposure to HDs (72 h) and HDACis (in the last 24 h of treatment), incubated alone or in combination. The effects of AZA or DEC, alone or in combination with HDACis are reported in panels A and B, respectively. Data are expressed as ΔCt (= Ct No Meth–Ct Meth), as means ± SEM. Statistical analysis: ANOVA + Tukey’s post-test. ***: *P* < 0.001.

**Fig 5:**
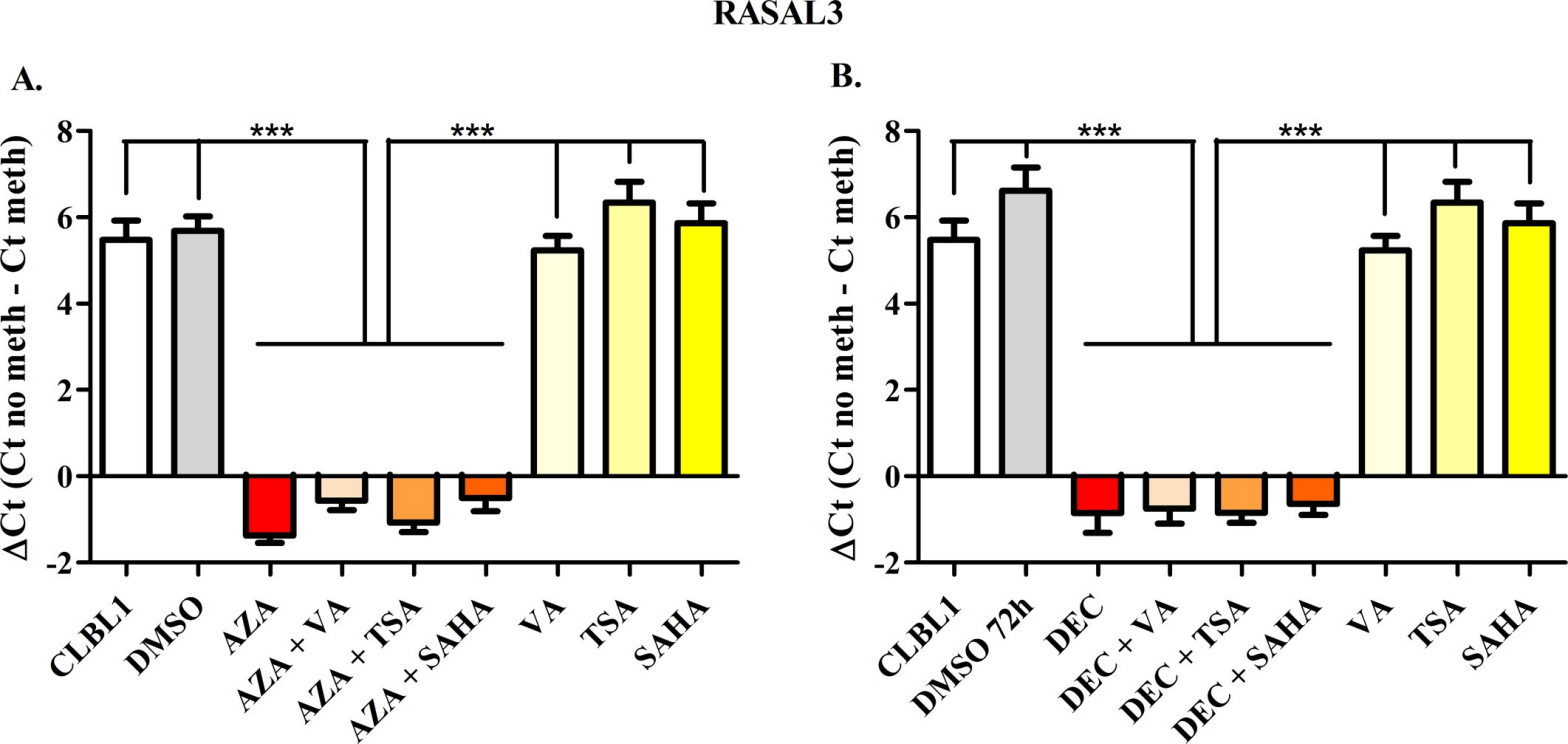
*RASAL3* Methyl Sensitive PCR after exposure to HDs (72 h) and HDACis (in the last 24 h of treatment), incubated alone or in combination. The effects of AZA or DEC, alone or in combination with HDACis are reported in panels A and B, respectively. Data are expressed as ΔCt (= Ct No Meth–Ct Meth), as means ± SEM. Statistical analysis: ANOVA + Tukey’s post-test. ***: *P* < 0.001.

**Fig 6:**
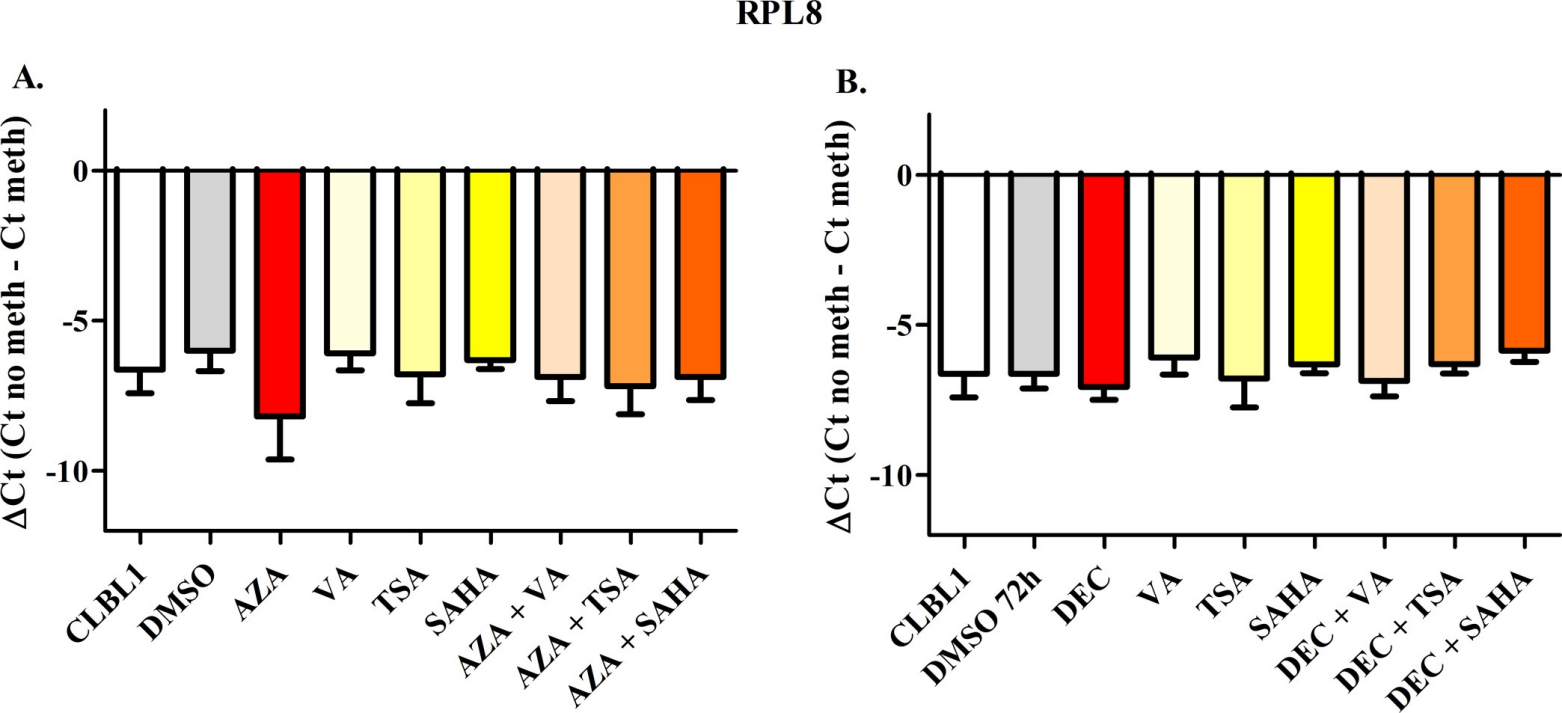
*RPL8* Methyl Sensitive PCR after exposure to HDs (72 h) and HDACis (in the last 24 h of treatment), incubated alone or in combination. The effects of AZA or DEC, alone or in combination with HDACis are reported in panels A and B, respectively. Data are expressed as ΔCt (= Ct No Meth–Ct Meth), as means ± SEM. Statistical analysis: ANOVA + Tukey’s post-test.

To semi-quantify the differential DNA methylation status between control and treated cells, the ΔCt value was calculated. Figs 2–6 show results obtained for *HOXD10*, *FGFR2*, *ITIH5*, *RASAL3*, and the negative control *RPL8*, respectively. Both AZA and DEC dramatically decreased *HOXD10*, *FGFR2*, *ITIH5* and *RASAL3* promoter methylation (*P* < 0.001). As expected, HDACis alone or in combination with HDs did not affect the promoter methylation of the four genes. No effects on the methylation status were observed for *RPL8* (negative control gene) in all treatment conditions (Fig 6).

### Gene expression in CLBL-1 cells

**Fig 7:**
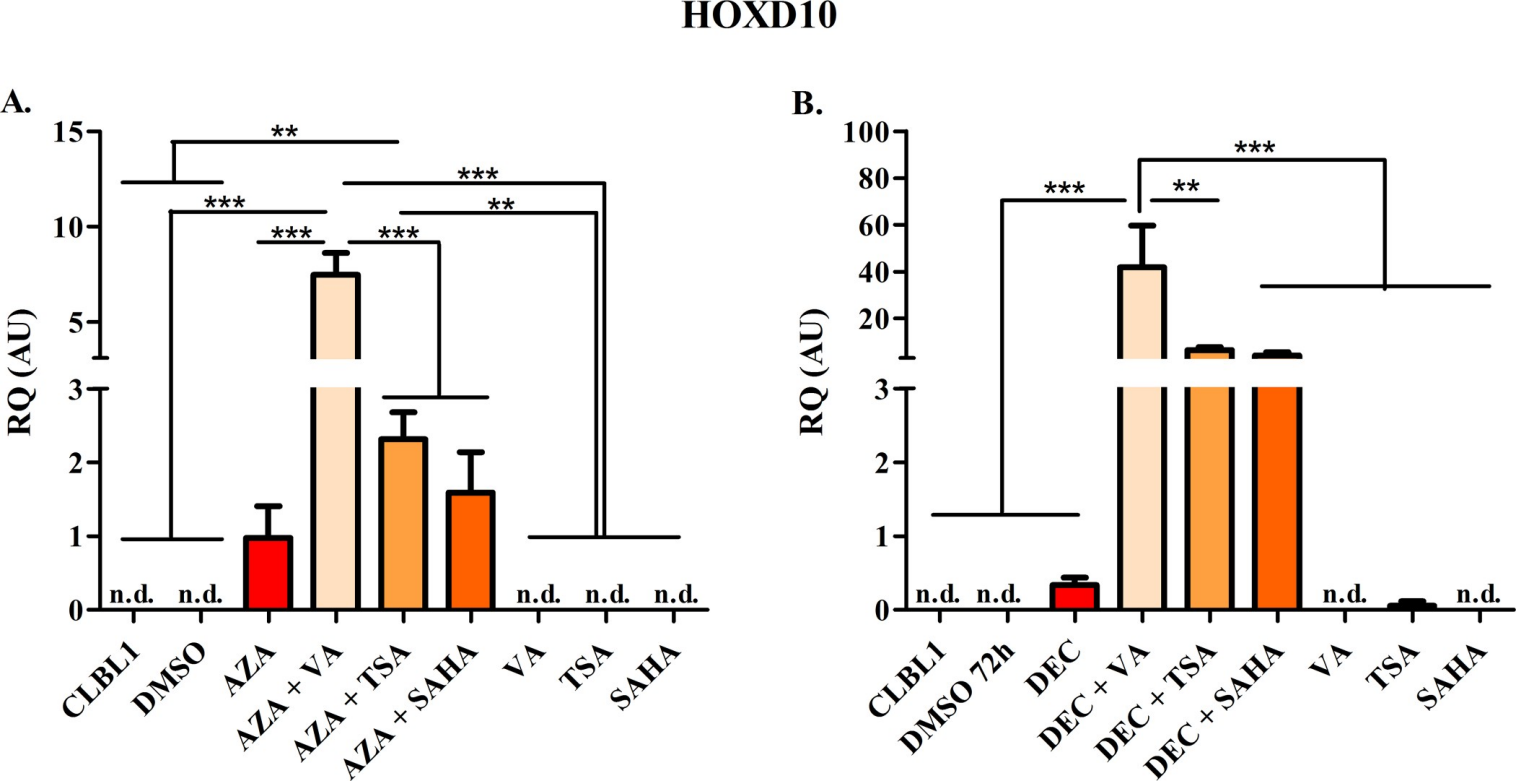
*HOXD10* mRNA re-expression following the exposure to HDs (72 h) and HDACis (in the last 24 h of treatment), alone or in combination. The effects of AZA or DEC, alone or in combination with HDACis, are reported in panel A and B, respectively. Data are expressed as Relative Quantification values (RQ), as means ± SEM. Statistical analysis: ANOVA + Tukey’s post-test. **: *P* < 0.01; ***: *P* < 0.001.—not detectable (n.d.).

**Fig 8:**
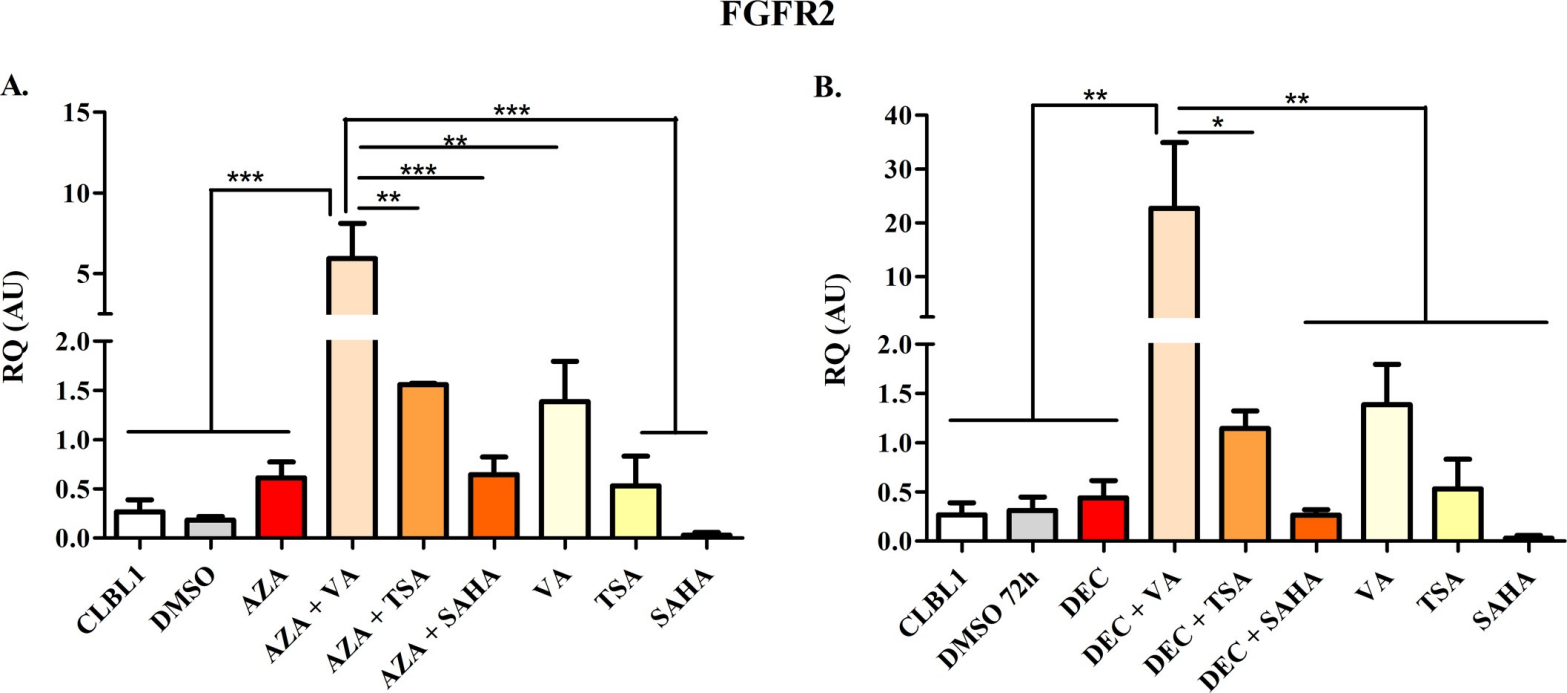
*FGFR2* mRNA re-expression following the exposure to HDs (72 h) and HDACis (in the last 24 h of treatment), alone or in combination. The effects of AZA or DEC, alone or in combination with HDACis, are reported in panel A and B, respectively. Data are expressed as Relative Quantification values (RQ), as means ± SEM. Statistical analysis: ANOVA + Tukey’s post-test. *: *P* < 0.05; **: *P* < 0.01; ***: *P* < 0.001.

**Fig 9:**
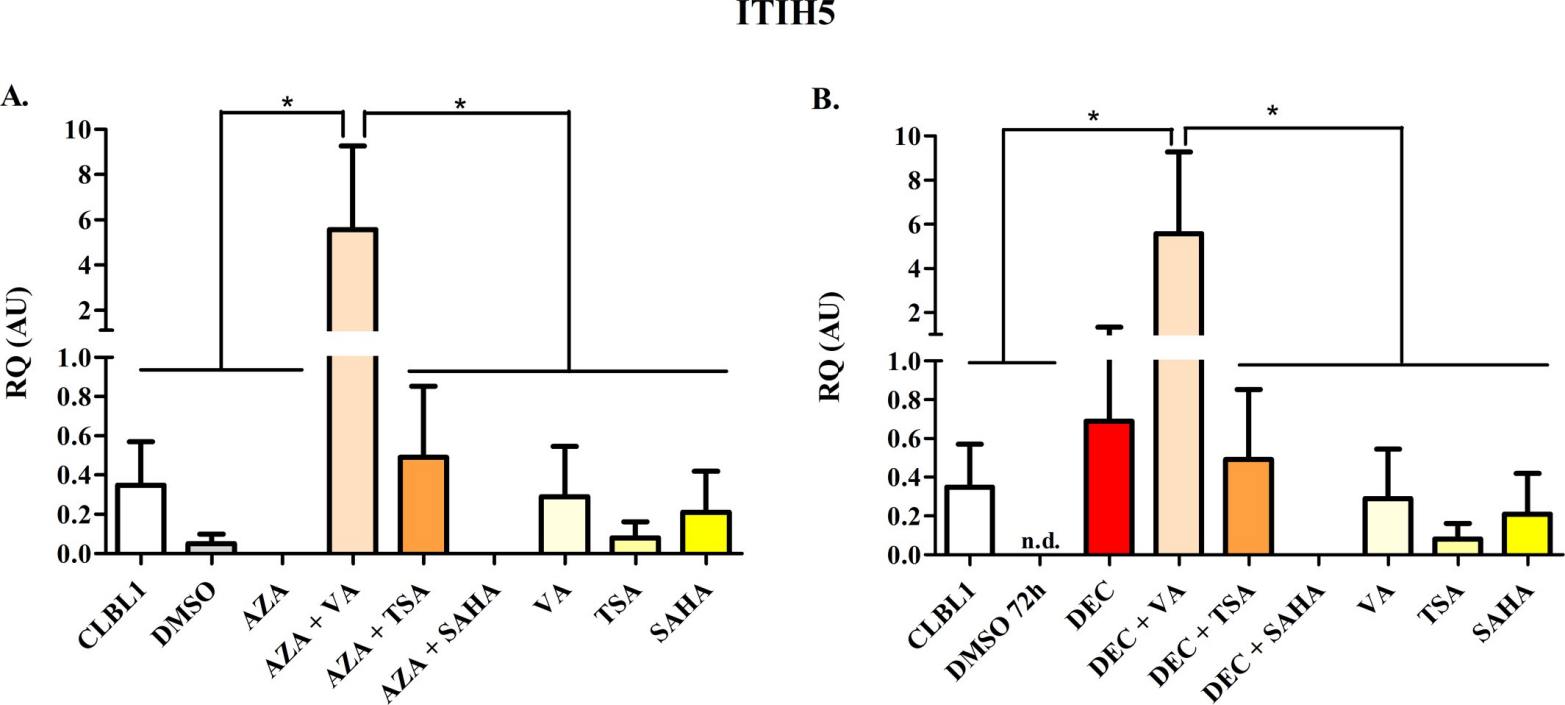
*ITIH5* mRNA expression after the treatment with HDs (72 h) and HDACis (in the last 24 h of incubation) alone or in combination. The effects of AZA or DEC, alone or in combination with HDACis, are reported in panel A and B, respectively. Data are expressed as Relative Quantification values (RQ), as means ± SEM. Statistical analysis: ANOVA + Tukey’s post-test. *: *P* < 0.05.

**Fig 10:**
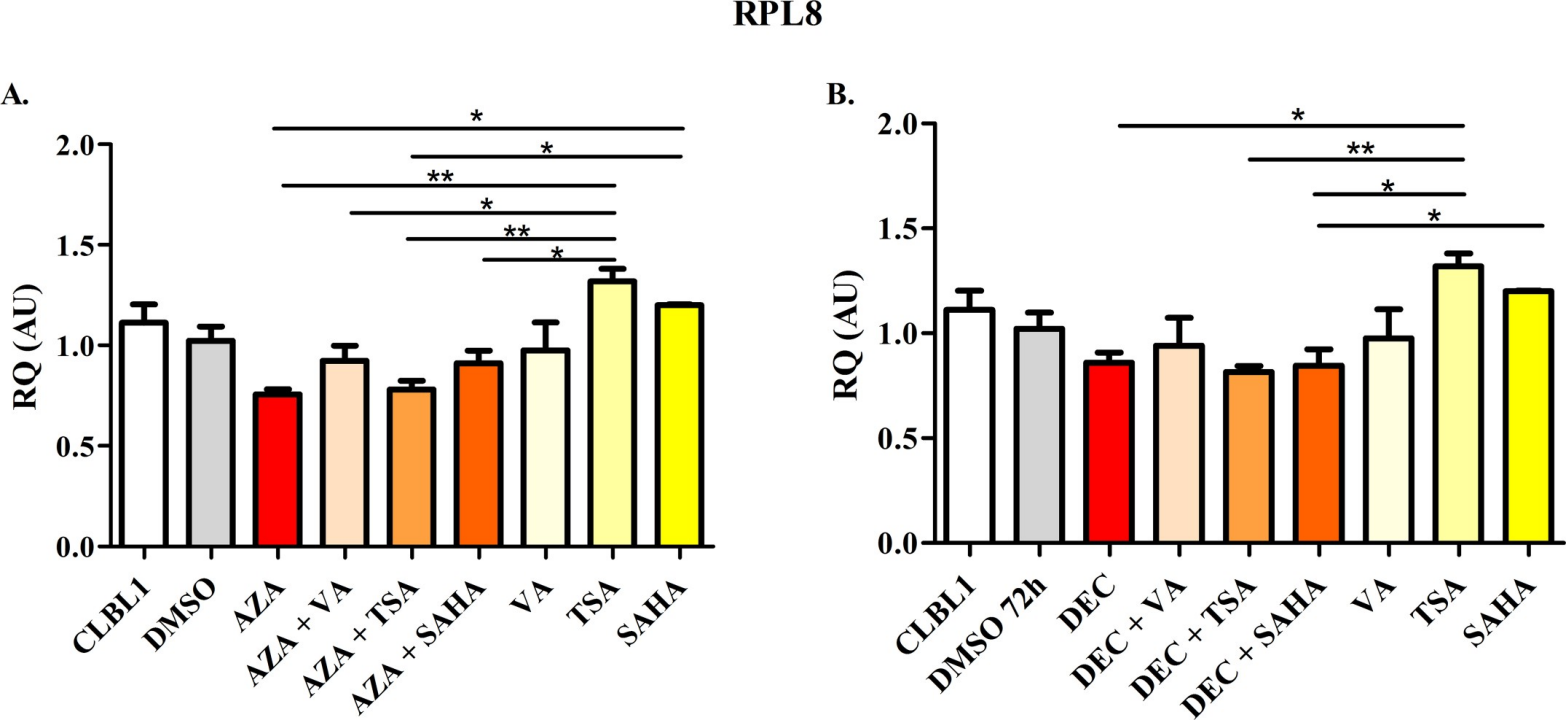
*RPL8* mRNA expression after the treatment with HDs (72 h) and HDACis (in the last 24 h of incubation) alone or in combination. The effects of AZA or DEC, alone or in combination with HDACis, are reported in panel A and B, respectively. Data are expressed as Relative Quantification values (RQ), as means ± SEM. Statistical analysis: ANOVA + Tukey’s post-test. *: *P* < 0.05; **: *P* < 0.01.

The effects of HDs and HDACis on mRNA expression of target genes *HOXD10*, *FGFR2*, and *ITIH5* as well as of *RPL8* (negative control) are summarized in Figs 7–10.

Basically, *HOXD10* mRNA was never detectable (n.d.) in untreated cells (Fig 7). Conversely, the treatment with HDs showed an increase of *HOXD10* mRNA levels, thereby allowing detection and quantification. A significant increase of *HOXD10* mRNA was noticed after AZA treatment (*P* < 0.05) when compared to CLBL-1 cells alone (unpaired *t*-test). Furthermore, AZA showed a higher effect than DEC on *HOXD10* re-expression. HDACis alone were not effective, while the combination HDs + HDACis caused a higher restoration (*P* < 0.01) when compared with HDs alone. Interestingly, the combination of DEC with HDACis resulted in a higher mRNA re-expression if compared to AZA + HDACis combination. A similar behaviour was observed with the association DEC + TSA and DEC + SAHA, whereas VA combined with both HDs elicited the highest reverting effect.

Scarce *FGFR2* mRNA levels were detected in control cells (Fig 8). AZA and DEC alone showed only a mild effect in re-expressing *FGFR2*. However, the reverting effect of HDACis alone (TSA and VA) was higher compared with AZA or DEC. The combination for both HDs and HDACis elicited a higher effect than the use of the single drug. This was evident for TSA and even more with VA (*P* < 0.01). SAHA alone or in association with HDs never showed statistically significant effects.

*ITIH5* is a rare transcript, showing a scarce and variable constitutive expression in CLBL-1 cells (Fig 9). Only the combination HDs + VA showed a significant restoration of gene expression. For *RASAL3* mRNA, unexpected results were obtained: specifically the treatment with both HDs and HDACis leaded to a significant inhibition of gene expression (S5 Fig).

The basal mRNA expression of the negative control *RPL8* recorded in control CLBL-1 cells was never affected by HDs and HDACis treatment, alone or in combination. A slight higher expression (about 1.2-fold vs DMSO) was observed only after TSA incubation (Fig 10).

### Effects of DEC on mice engrafted with CLBL-1 cells

Eight NOD-Scid mice were engrafted with CLBL-1 cells and treated with DEC when tumours volume was 800 mm^3^ in average. Overall, no observable adverse effects were ever noticed during the treatment. DEC alone failed to arrest the tumour growth; in particular, xeno-tumours were not affected by the treatment even when the dose of DEC was doubled (4 mg/Kg, i.p. administered on days 2, 3, 4). No statistically significant differences were achieved in tumour size between DEC treated mice and control mice receiving vehicle only. However, a slighter reduction of growth was observed in treated mice (S6 Fig). Furthermore, spleen infiltration was not observed in mice receiving vehicle.

**Fig 11:**
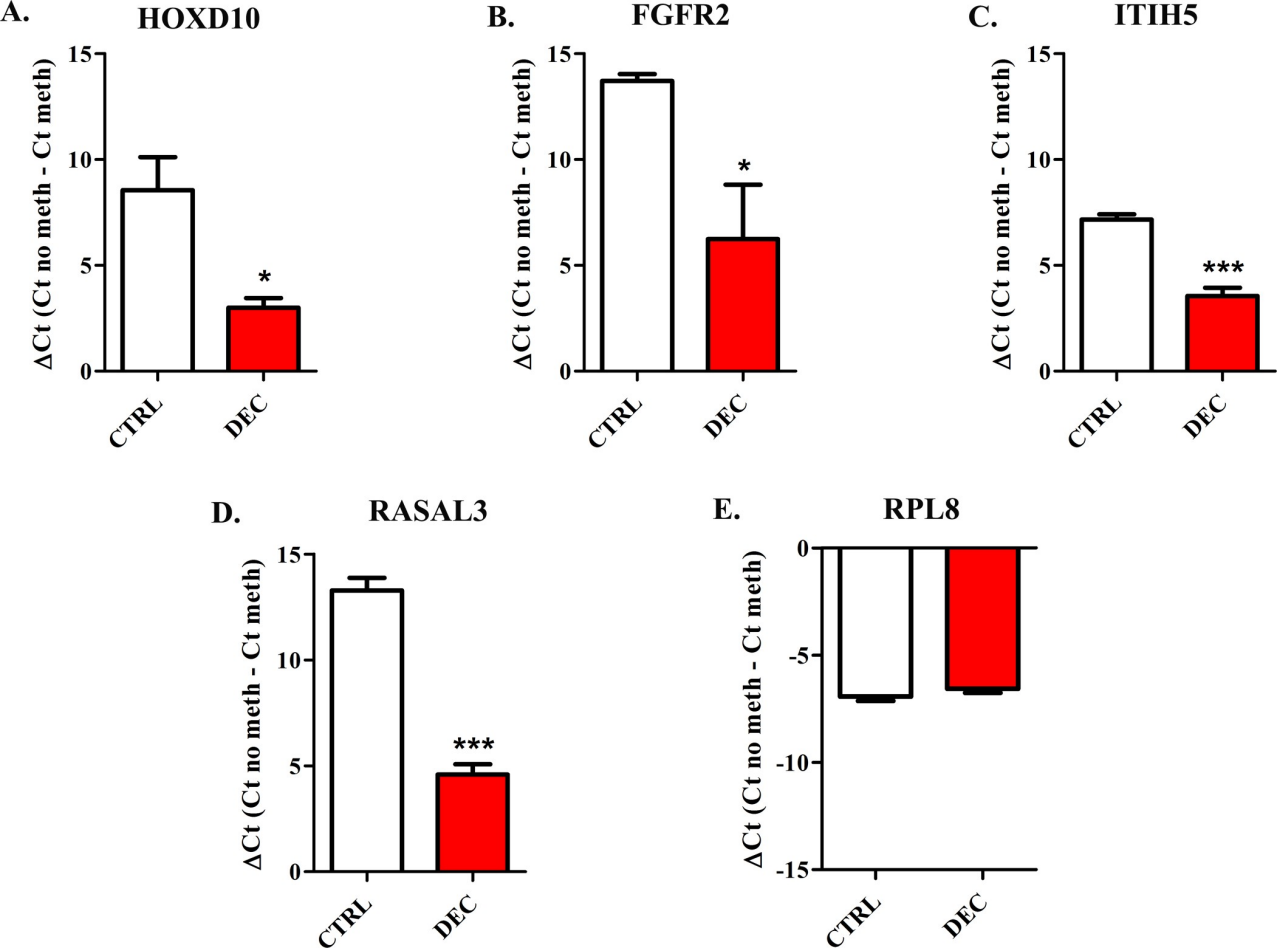
Methyl Sensitive PCR in Nod-Scid mice engrafted with CLBL-1 cells and treated with DEC or vehicle. Effects of DEC treatment in xeno-tumours for *HOXD10* (A), *FGFR2* (B), *ITIH5* (C), *RASAL3* (D) and *RPL8* (E). Data are expressed as ΔCt (= Ct No Meth–Ct Meth), as means ± SEM. Statistical analysis: unpaired T test. *: *P* < 0.05, ***: *P* < 0.001.

When MSP was applied to tumour samples, a significant reduction of *HOXD10*, *FGFR2* (*P* < 0.05), *ITIH5* and *RASAL3* (*P* < 0.001) promoter methylation was observed in DEC-treated mice (Fig 11). The gene expression was not affected by the treatment. As shown in S7 Fig, only a slight re-expression of *HOXD10* mRNA was observed in DEC-treated mice, while *RPL8* was not modulated by the treatment, as expected. The other target genes considered in the present study were not quantifiable in both control and treated tumours.

## Discussion

Based on previous observations of methylation changes in canine DLBCL [29], the present study aimed to corroborate, using *in vitro* and *in vivo* approaches, the promoter methylation of four TSGs (*HOXD10*, *FGFR2*, *ITIH5* and *RASAL3*), whose mRNA expression was confirmed to be significantly reduced in DLBCL samples vs controls (S1 Fig). To this purpose we evaluated the effects of two classes of epigenetic drugs, HDs and HDACis using CLBL-1 cells, representing the unique canine BCL cell line available in veterinary medicine. A similar *in vitro* approach was previously considered in dogs, but a preliminary cytotoxicity screening, supporting the chosen HD concentrations, was not executed [21–23]. Herein, we determined the HDs cytotoxicity before conducting further experiments.

Besides methylation, other epigenetic mechanisms are likely to contribute to gene silencing, such as histone deacetylation. To comprehend the role of deacetylation/acetylation balance in reverting gene expression in our cellular model, HDACis alone or in combination with HDs were considered; this represents an innovative element of this work. HDACis were selected according to available dog pharmacokinetics and pharmacodynamics data [23, 27, 36–41]. After IC_50_ values determination, supplementary experiments were performed to obtain the best drug concentration taking into consideration toxicity and biological effects (data not shown). Finally, IC_50_ values for AZA and DEC and IC_20_ values for TSA, SAHA and VA were considered for the subsequent experiments.

Afterwards, we assessed the ability of HDs and HDACis to reactivate the expression of our four candidate genes, known to be methylated at the promoter region [29] and inhibited (S1 Fig) in canine DLBCL. These targets play a putative role as TSG in humans and possess an epigenetic mechanism of regulation in human cancer [42–51]. AZA and DEC dramatically decreased the promoter methylation of *HOXD10*, *FGFR2*, *ITIH5* and *RASAL3*, although showing a gene-specific effect, as previously reported [52]. Conversely, HDACis alone did not affect the methylation status of target genes in control cells; moreover, their combination with HDs did not show additive effects. These results indirectly support the evidence that all the genes were hypermethylated at basal conditions in CLBL-1 cells, and treatment conditions with HDs were able to revert their initial methylation status. Furthermore, the absence of HDs and HDACis effects on *RPL8* methylation demonstrates that these effects were specific for BCL associated hypermethylated TSGs and were not widespread to other targets.

To demonstrate the epigenetic-dependent silencing of these genes, mRNA expression was evaluated, too. *HOXD10* was not expressed in untreated CLBL-1 cells. In our experimental conditions, AZA and DEC, when used alone, were able to restore *HOXD10* expression, completely silenced in control CLBL-1 cells before treatment. Conversely, HDACis modulated mRNA expression only when used in combination. This evidence indirectly supports the hypothesis that histone deacetylation might play an important role in regulating gene expression. On top, the highest mRNA re-expression of *HOXD10* was observed with the combination DEC + HDACis, confirming previous published data [43].

*FGFR2* was expressed at very low levels in untreated cells. Both AZA and DEC used alone showed a mild effect in re-expressing *FGFR2*, whereas the combination of HDs and HDACis a higher effect. These data might suggest the potential contribution of histone modifications on *FGFR2* silencing, as already described in a previous work in which TSA reverted *FGFR2* expression in mouse AtT20 (pituitary corticotropic tumour) cells and acetylation/deacetylation balance was identified as the driver mechanism in the epigenetic regulation of this TSG [48]. Nevertheless, being out of the scope of the present paper, the acetylation state of *FGFR2* and the other selected target loci was not measured in CLBL-1 cells before and after the treatment with HDACis. Thus, the contribution of acetylation/deacetylation balance in the control of gene expression was not specifically assessed. Worth mentioning, since HDACIs here used are both histone and nonhistone substrates, they could regulate gene expression through the acetylation of other proteins, including transcription factors [53]; consequently, the observed effects on the gene expression could be attributable to further molecular mechanisms than histone acetylation/deacetylation.

Similarly to *FGFR2*, *ITIH5* was also very poorly expressed in untreated cells. Only the combination of HDs and VA showed a significant restoration of gene expression, in accordance with former published data [46, 47]. Higher fold-changes have been reported, but using higher concentrations of DEC and TSA [54]; however, in our experimental conditions higher concentrations caused cell death and subsequently the modulation of gene expression could not be measured (data not shown). Once more, our results highlight gene- and also cell-specific effects of both HDs and HDACis [52].

Despite the *RASAL3* inhibition in DLBCLs compared to controls, the exposure to HDs and HDACis, either as single agent than in combination, did not increase *RASAL3* gene expression in CLBL-1 cells. Both AZA and DEC reduced the promoter methylation but did not induce mRNA expression. Despite the observed incongruence between MSP and gene expression data, this result is worth of mention considering the scarce information available for this gene. Hence, further studies are needed to better understand the effect of methylation on gene transcription and, consequently, the role of *RASAL3* in the proliferation, development and biological activity of B-cells [51, 55].

As a whole, our results support the hypothesis that CLBL-1 cells represent a reliable *in vitro* model for studying epigenetic modifications in canine BCL. However, the methodological approach here used showed some limitations, as the measurement of the methylation status of only one regulatory element for each gene (although corresponding to DNA regions recognized by the probes of the canine Agilent CpG microarray platform used in our previous study); the lack of histone acetylation measurement; lastly, the absence of functional studies useful to define the role of target genes on cellular homeostasis, survival or proliferation in canine BCL.

Nevertheless, we decided to further confirm results *in vivo*, using a murine model. In this respect, NOD-Scid mice were engrafted with CLBL-1 cells and treated with DEC. The choice of DEC as single agent was based on a previous experience, in which in a model of human Splenic Marginal Zone lymphoma, established by engrafting SSK41 cells in Nod-Scid mice, DEC was used at the dose of 2 mg/Kg, given twice starting when tumours were 100 mm^3^ (Day 1 and Day 3). Using this treatment schedule, DEC was able to eradicate tumours in three weeks (data not shown). In our study, DEC treatment started late because we were interested to obtain tumour samples for molecular and histological analysis. Unfortunately, DEC treatment was unsuccessful in these experimental conditions. However, it significantly decreased the methylation of *HOXD10*, *FGFR2*, *ITIH5* and *RASAL3* promoters. A hypothesis beyond this clinical failure might be the extreme biological aggressiveness of CLBL-1 cell line, as demonstrated by the short doubling time [28]. Due to toxicity and ethical issues, it was not possible to use higher DEC concentrations or further prolong treatments. This experiment highlights the inefficiency of DEC used as a single agent and confirms the use of a combined treatment with HDACis or CHOP regimen for maximizing the therapeutic effect, as reported by other authors [52, 56–59].

## Conclusions

In summary, the findings of this paper demonstrate that the four genes here assayed are biomarkers of hypermethylation in canine DLBCL. Specifically, the treatment of CLBL-1 cell line with HDs reduces methylation levels, and their combination with HDACis significantly increase the mRNA expression of three silenced genes. In perspectives, specific mechanistic and functional studies will be performed to deepen the methylation-dependent mechanisms of gene silencing and comprehend the pathogenic role of these same genes, evaluating their potential contribution to cell survival and proliferation following re-expression. Furthermore, the CLBL-1 cell line, a reliable *in vitro* model for studying epigenetics in canine BCL, has been proved useful also for the obtainment of xenograft DLBCL tumours in mice, even though the tumour volume at time of treatment and treatment regimen need further optimization.

## Supporting information

S1 Checklist Da Ros_ARRIVE guidelines checklist. (PDF)

S1 Table Concentrations of Meth and No Meth primer pairs used in methyl specific PCR (MSP) analysis. (PDF)

S2 Table Sequences of primer pairs used in gene expression analysis. (PDF)

S3 Table Main features (F and R primer concentration, slope, efficiency, R^2^, dynamic range) of qPCR assays. (PDF)

S1 Fig *HOXD10*, *FGFR2*, *ITIH5*, *RASAL3* and *RPL8* mRNA expression in canine control lymph nodes and DLBCL samples. **A.** *HOXD10*, **B.** *FGFR2*, **C.** *ITIH5*, **D.** *RASAL3*, **E.** *RPL8*. The mRNA expression of target and negative control genes was measured in 11 control lymph nodes and 12 DLBCL samples using qPCR assays reported in the Material and Methods section. Relative quantification values (RQ) are expressed as mean ± SEM. Statistical analysis: Mann Whitney test. *: *P* < 0.05, **: *P* < 0.01; ***: *P* < 0.001.
(PDF)

S2 Fig 2% agarose gel electrophoresis of PCR products obtained using *HOXD10* and *FGFR2* Meth and No-Meth primers. Panel A. *HOXD10*, Panel B. *FGFR2*. 1. Meth primer gDNA bisulfite converted; 2. Meth primer gDNA non-bisulfite converted; 3. Meth primer blank ctrl; 4. No Meth primer gDNA bisulfite converted; 5. No Meth primer gDNA non-bisulfite converted; 6. No Meth primer blank ctrl.
(PDF)

S3 Fig **Melting curve of *HOXD10* Meth (a.) and No Meth (b.) primers.** ― blank control; ― non-bisulfite converted gDNA; ― bisulfite-converted gDNA; ― bisulfite-converted gDNA from CLBL1 treated with AZA + VA.
(PDF)

S4 Fig **Melting curve of *FGFR2* Meth (a.) and No Meth (b.) primers.** ― blank control; ― non-bisulfite converted gDNA; ― bisulfite-converted gDNA; ― bisulfite-converted gDNA from CLBL-1 treated with AZA + VA.
(PDF)

S5 Fig *RASAL3* mRNA re-expression following the exposure to HDs (72 h) and HDACis (in the last 24 h of treatment), alone or in combination. The effects of AZA or DEC, alone or in combination with HDACis, are reported in panel A and B, respectively.
(PDF)

S6 Fig Tumour size in control and DEC treated mice. NOD-Scid mice were subcutaneously engrafted with 15x10^6^ CLBL-1 cells and at an average tumour volume of 800 mm^3^ were treated with DEC (n = 4) or vehicle only (n = 4). DEC was administered four times at the dose of 2 mg/kg (Day 1) and 4 mg/kg (Days 2, 3, 4) through i.p. injections. At the sacrifice, tumours transplanted were collected and measured in size.
(PDF)

S7 Fig *HOXD10* and *RPL8* mRNA expression in Nod-Scid mice engrafted with CLBL-1 cells and treated with DEC or vehicle. Effects of DEC treatment on *HOXD10* (A) and *RPL8* (B) mRNA expression in xenograft tumours. Statistical analysis: unpaired T test.
(PDF)
