# Supplementary material for: Validation of epigenetic mechanisms regulating gene expression in canine B-cell lymphoma: An in vitro and in vivo approach
Source: PLoS One. 2018 Dec 11;13(12):e0208709. doi: 10.1371/journal.pone.0208709 (PMC6289462; doi:10.1371/journal.pone.0208709)
Supplement: S1 Table — (PDF) [file pone.0208709.s002.pdf]

S1 Table. Concentrations of Meth and No Meth primer pairs used in methyl specific PCR (MSP) analysis.

Gene	Primer Meth F/R (nM)	Primer No Meth F/R (nM)
HOXD10	600/600	600/600
FGFR2	600/600	600/600
ITIH5	300/600	600/600
RASAL3	50/50	50/50
RPL8	50/50	50/300
