# Supplementary material for: Validation of epigenetic mechanisms regulating gene expression in canine B-cell lymphoma: An in vitro and in vivo approach
Source: PLoS One. 2018 Dec 11;13(12):e0208709. doi: 10.1371/journal.pone.0208709 (PMC6289462; doi:10.1371/journal.pone.0208709)
Supplement: S2 Table — (PDF) [file pone.0208709.s003.pdf]

S2 Table. Sequences of primer pairs used in gene expression analysis.

Gene	NCBI or ENSEMBL transcript ID		Sequence 5'- 3'	References
HOXD10	XM_005640343.1	Forward	CGTGTCCAGTCCCGAAGTAC	designed *ex novo*
		Reverse	GCCAATTGCTGGTTGGAGTATC	
FGFR2	NM_001003336.1	Forward	GAAGCGGTGGGAATTGACAAA	designed ex novo
		Reverse	GATCTTTCTCTGTGGCATCGTC	
ITIH5	XM_005617202.1	Forward	GGAGGTGCTGCCCTTACAG	designed ex novo
		Reverse	GCGAAAGTGTCGTTTTGGTTGA	
RASAL3	XM_005632762.1	Forward	CCCTCTGGGGAAAACATAAGA	designed ex novo
		Reverse	TCCAGATGGGTACGTCAGG	
RPL8	ENSCAFT00000002627	Forward	GGACGGAGCTGTTCATCG	Giantin et al. (2016)*
		Reverse	GCACATTGCCTATGTTGAGC	

* Giantin M, Baratto C, Marconato L, Vascellari M, Mutinelli F, Dacasto M et al. Transcriptomic analysis identified up-regulation of a solute carrier transporter and UDP glucuronosyltransferases in dogs with aggressive cutaneous mast cell tumours. The Veterinary Journal 2016; 212: 36-43.
