# Supplementary material for: Validation of epigenetic mechanisms regulating gene expression in canine B-cell lymphoma: An in vitro and in vivo approach
Source: PLoS One. 2018 Dec 11;13(12):e0208709. doi: 10.1371/journal.pone.0208709 (PMC6289462; doi:10.1371/journal.pone.0208709)
Supplement: S3 Table — (PDF) [file pone.0208709.s004.pdf]

S3 Table. Main features (F and R primer concentration, slope, efficiency, R^2, dynamic range) of qPCR assays.

Gene	F/R (nM)	Slope	Efficiency (%)	R^2	Dynamic range (Ct)
HOXD10	50/300	-3.32	100.1	0.99	29.88 - 37.15
FGFR2	50/300	-3.44	95.3	0.99	32.44 – 39.43
ITIH5	300/50	-3.40	97.1	0.99	24.54 – 38.82
RASAL3	50/50	-3.31	100.4	0.98	26.93 – 34.18
RPL8	300/300	-3.19	106.0	0.99	14.73 – 23.81
GOLGA1	300/300	-3.17	106.6	0.99	25.01 – 33.50
CCZ1	600/600	-3.21	105.1	0.99	20.95 – 30.05
