# Supplementary material for: Validation of epigenetic mechanisms regulating gene expression in canine B-cell lymphoma: An in vitro and in vivo approach
Source: PLoS One. 2018 Dec 11;13(12):e0208709. doi: 10.1371/journal.pone.0208709 (PMC6289462; doi:10.1371/journal.pone.0208709)

S1 Fig. *HOXD10, FGFR2, ITIH5, RASAL3* and *RPL8* mRNA expression in canine control lymph nodes and DLBCL samples. A. *HOXD10*, **B.** *FGFR2*, **C.** *ITIH5*, **D.** *RASAL3*, **E.** *RPL8*. The mRNA expression of target and negative control genes was measured in 11 control lymph nodes and 12 DLBCL samples using qPCR assays reported in the Material and Methods section. Relative quantification values (RQ) are expressed as mean ± SEM. Statistical analysis: Mann Whitney test. *: $P < 0.05$, **: $P < 0.01$; ***: $P < 0.001$.

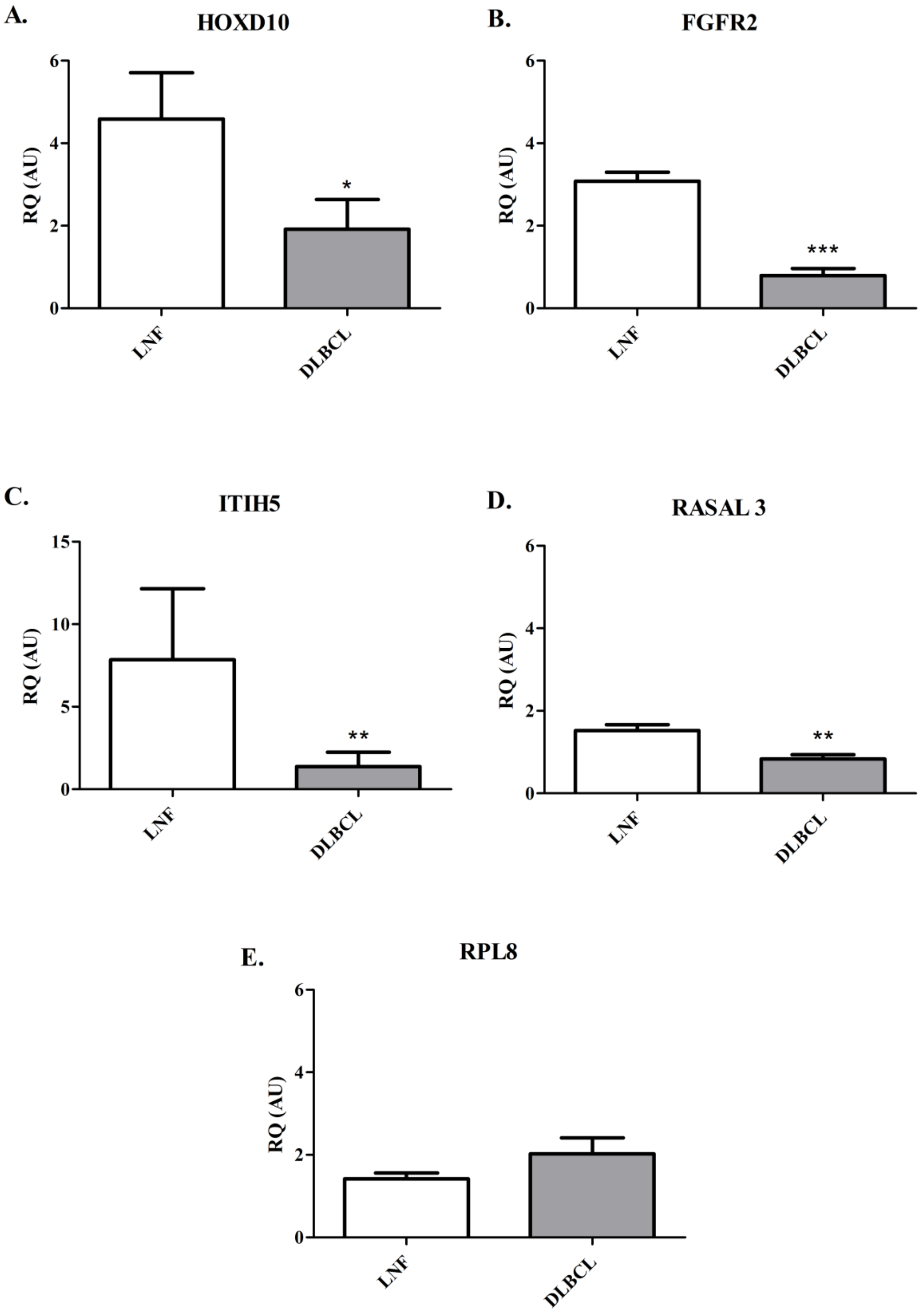

Supplement: S1 Fig — A. HOXD10, B. FGFR2, C. ITIH5, D. RASAL3, E. RPL8. The mRNA expression of target and negative control genes was measured in 11 control lymph nodes and 12 DLBCL samples using qPCR assays reported in the Material and Methods section. Relative quantification values (RQ) are expressed as mean ± SEM. Statistical analysis: Mann Whitney test. *: P < 0.05, **: P < 0.01; ***: P < 0.001. (PDF) [file pone.0208709.s005.pdf]
