# Supplementary material for: Validation of epigenetic mechanisms regulating gene expression in canine B-cell lymphoma: An in vitro and in vivo approach
Source: PLoS One. 2018 Dec 11;13(12):e0208709. doi: 10.1371/journal.pone.0208709 (PMC6289462; doi:10.1371/journal.pone.0208709)

S2 Fig. 2% agarose gel electrophoresis of PCR products obtained using *HOXD10* and *FGFR2* Meth and No-Meth primers. Panel A. *HOXD10*, **Panel B.** *FGFR2*. 1. Meth primer gDNA bisulfite converted; 2. Meth primer gDNA non-bisulfite converted; 3. Meth primer blank ctrl; 4. No Meth primer gDNA bisulfite converted; 5. No Meth primer gDNA non-bisulfite converted; 6. No Meth primer blank ctrl.

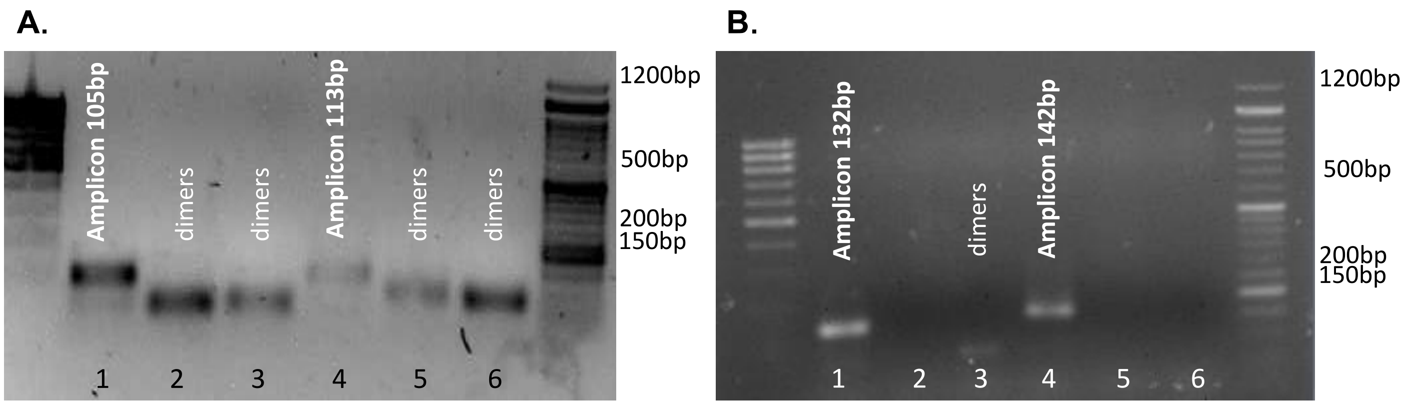

Supplement: S2 Fig — Panel A. HOXD10, Panel B. FGFR2. 1. Meth primer gDNA bisulfite converted; 2. Meth primer gDNA non-bisulfite converted; 3. Meth primer blank ctrl; 4. No Meth primer gDNA bisulfite converted; 5. No Meth primer gDNA non-bisulfite converted; 6. No Meth primer blank ctrl. (PDF) [file pone.0208709.s006.pdf]
