# Supplementary material for: Validation of epigenetic mechanisms regulating gene expression in canine B-cell lymphoma: An in vitro and in vivo approach
Source: PLoS One. 2018 Dec 11;13(12):e0208709. doi: 10.1371/journal.pone.0208709 (PMC6289462; doi:10.1371/journal.pone.0208709)

**S4 Fig. Melting curve of *FGFR2* Meth (a.) and No Meth (b.) primers.** — blank control; — non-bisulfite converted gDNA; — bisulfite-converted gDNA; — bisulfite-converted gDNA from CLBL-1 treated with AZA + VA.

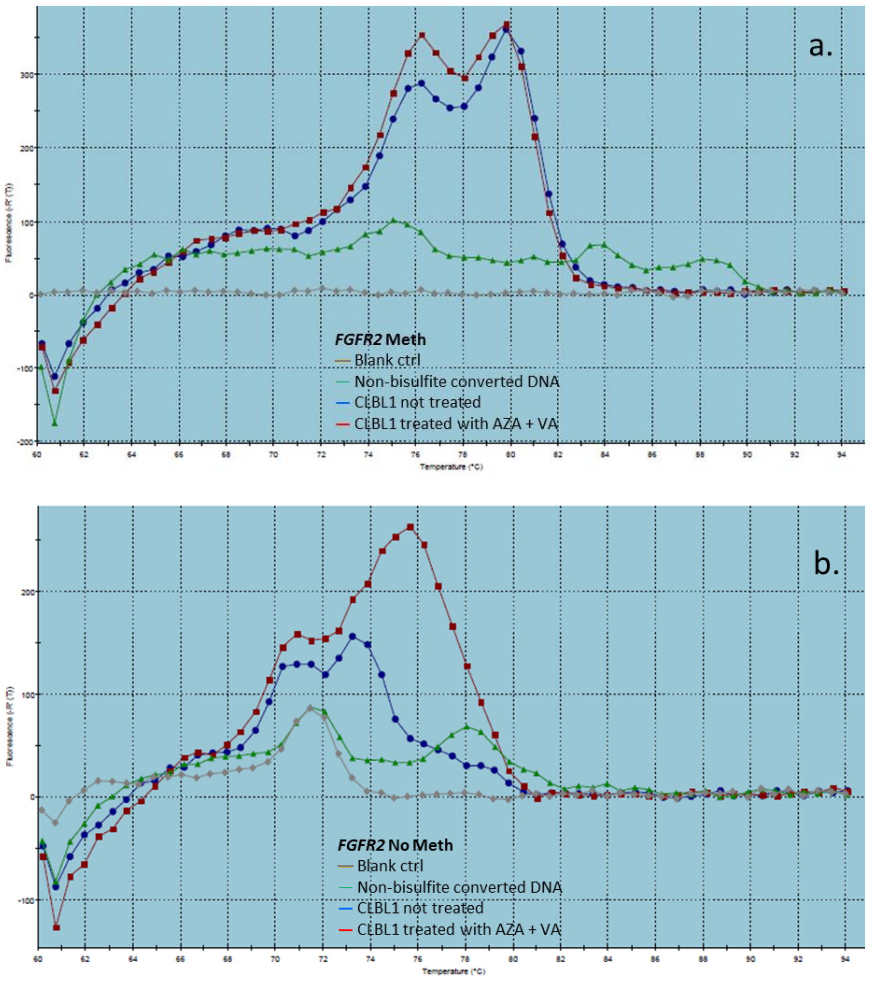

Supplement: S4 Fig — Melting curve of FGFR2 Meth (a.) and No Meth (b.) primers. ― blank control; ― non-bisulfite converted gDNA; ― bisulfite-converted gDNA; ― bisulfite-converted gDNA from CLBL-1 treated with AZA + VA. (PDF) [file pone.0208709.s008.pdf]
