# Supplementary material for: Validation of epigenetic mechanisms regulating gene expression in canine B-cell lymphoma: An in vitro and in vivo approach
Source: PLoS One. 2018 Dec 11;13(12):e0208709. doi: 10.1371/journal.pone.0208709 (PMC6289462; doi:10.1371/journal.pone.0208709)

S5 Fig. *RASAL3* mRNA expression following the exposure to HDs (72 h) and HDACis (in the last 24 h of treatment), alone or in combination. The effects of AZA or DEC, alone or in combination with HDACis, are reported in panel A and B, respectively.

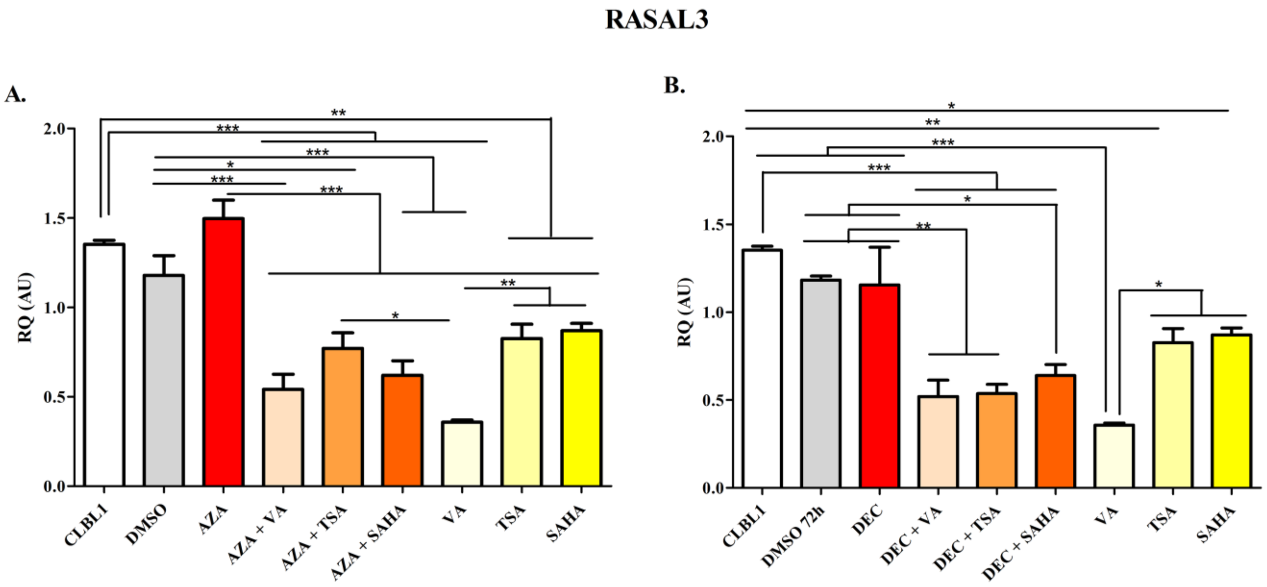

Supplement: S5 Fig — The effects of AZA or DEC, alone or in combination with HDACis, are reported in panel A and B, respectively. (PDF) [file pone.0208709.s009.pdf]
