# Supplementary material for: Validation of epigenetic mechanisms regulating gene expression in canine B-cell lymphoma: An in vitro and in vivo approach
Source: PLoS One. 2018 Dec 11;13(12):e0208709. doi: 10.1371/journal.pone.0208709 (PMC6289462; doi:10.1371/journal.pone.0208709)

S6 Fig. Tumour size in control and DEC treated mice. NOD-Scid mice were subcutaneously engrafted with $15x10^6$ CLBL-1 cells and at an average tumour volume of 800 mm^3 were treated with DEC (n = 4) or vehicle only (n = 4). DEC was administered four times at the dose of 2 mg/kg (Day 1) and 4 mg/kg (Days 2, 3, 4) through i.p. injections. At the sacrifice, tumours transplanted were collected and measured in size (mm^3).

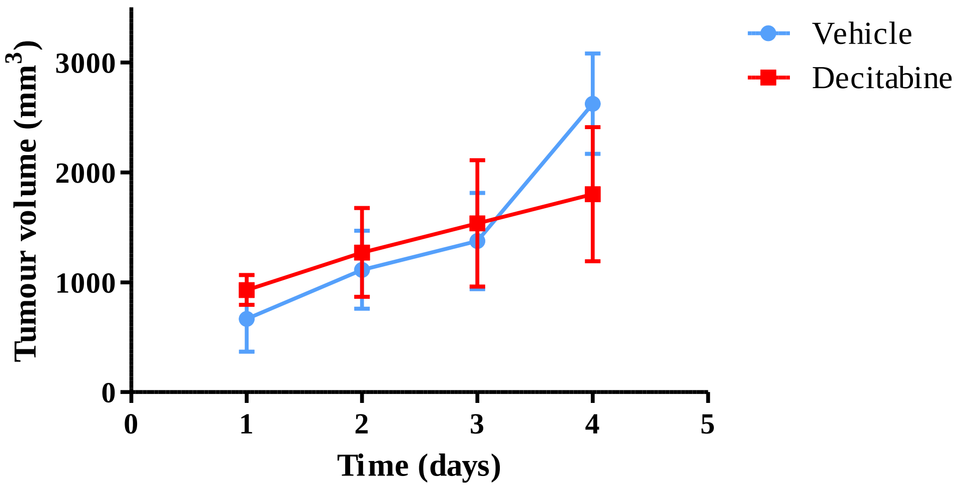

Supplement: S6 Fig — NOD-Scid mice were subcutaneously engrafted with 15x106 CLBL-1 cells and at an average tumour volume of 800 mm3 were treated with DEC (n = 4) or vehicle only (n = 4). DEC was administered four times at the dose of 2 mg/kg (Day 1) and 4 mg/kg (Days 2, 3, 4) through i.p. injections. At the sacrifice, tumours transplanted were collected and measured in size. (PDF) [file pone.0208709.s010.pdf]
