# Supplementary material for: Validation of epigenetic mechanisms regulating gene expression in canine B-cell lymphoma: An in vitro and in vivo approach
Source: PLoS One. 2018 Dec 11;13(12):e0208709. doi: 10.1371/journal.pone.0208709 (PMC6289462; doi:10.1371/journal.pone.0208709)

S7 Fig. *HOXD10* and *RPL8* mRNA expression in Nod-Scid mice engrafted with CLBL-1 cells and treated with DEC or vehicle. Effects of DEC treatment on *HOXD10* (A) and *RPL8* (B) mRNA expression in xenograft tumours. Statistical analysis: unpaired T test.

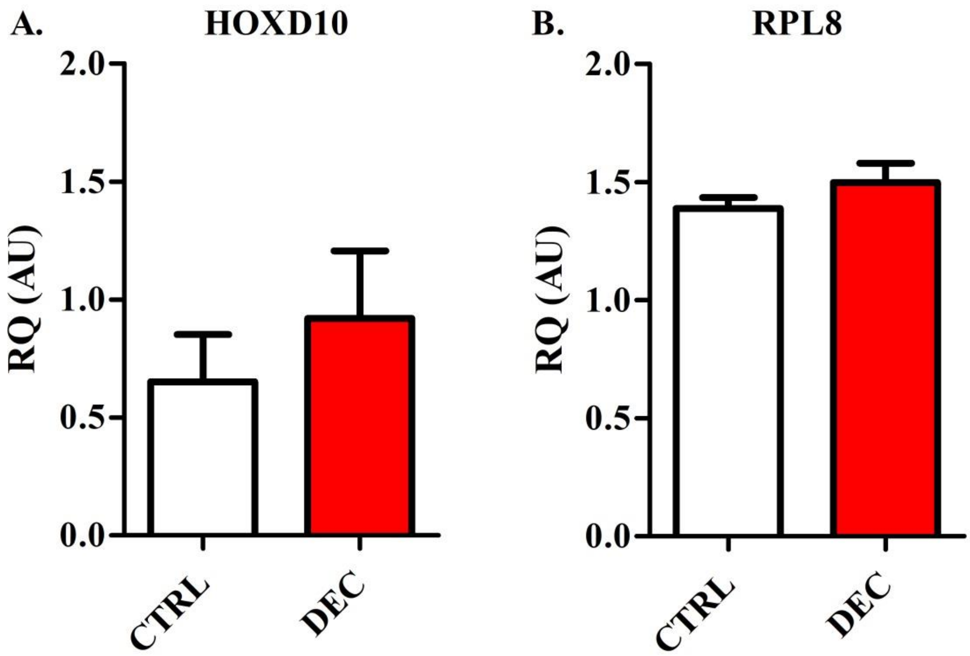

Supplement: S7 Fig — Effects of DEC treatment on HOXD10 (A) and RPL8 (B) mRNA expression in xenograft tumours. Statistical analysis: unpaired T test. (PDF) [file pone.0208709.s011.pdf]
